# Impact of cavotricuspid isthmus depth on the ablation index for successful first-pass typical atrial flutter ablation

**DOI:** 10.1038/s41598-021-01846-7

**Published:** 2021-11-17

**Authors:** Manabu Kashiwagi, Akio Kuroi, Yosuke Katayama, Kosei Terada, Suwako Fujita, Takeshi Hozumi, Kunihiro Shimamura, Yasutsugu Shiono, Takashi Tanimoto, Takashi Kubo, Atsushi Tanaka, Takashi Akasaka

**Affiliations:** 1 Department of Cardiovascular Medicine, Wakayama Medical University, 811-1, Kimiidera, Wakayama City, Wakayama 641-8509 Japan; 2 Department of Cardiovascular Medicine, Shingu Municipal Medical Center, 18-7, Hachibuse, Shingu, Wakayama 647-0072 Japan

**Keywords:** Interventional cardiology, Arrhythmias

## Abstract

Cavotricuspid isthmus (CTI) linear ablation has been established as the treatment for typical atrial flutter. Recently, ablation index (AI) has emerged as a novel marker for estimating ablation lesions. We investigated the relationship between CTI depth and ablation parameters on the procedural results of typical atrial flutter ablation**.** A total of 107 patients who underwent CTI ablation were retrospectively enrolled in this study. All patients underwent computed tomography before catheter ablation. From the receiver-operating curve, the best cut-off value of CTI depth was < 4.1 mm to predict first-pass success. Although the average AI was not different between deep CTI (DC; CTI depth ≥ 4.1) and shallow CTI (SC; CTI depth < 4.1), DC required a longer ablation time and showed a lower first-pass success rate (*p* < 0.01). In addition, the catheter inversion technique was more frequently required in the DC (*p* < 0.01). The lowest AI sites of the first-pass CTI line were determined in both the ventricular (2/3 segment of CTI) and inferior vena cava (IVC, 1/3 segment of CTI) sides. The best cut-off values of the weakest AIs at the ventricular and IVC sides for predicting first-pass success were > 420 and > 386, respectively. Among patients with these cut-off values, the first-pass success rate was 89% in the SC and 50% in the DC (*p* < 0.01). Although ablation parameters were not significantly different, the first-pass success rate was lower in the DC than in the SC. Further investigation might be required for better outcomes in deep CTIs.

## Introduction

Cavotricuspid isthmus (CTI) linear ablation has been established as the treatment for typical atrial flutter (AFL)^1,2^. Although the short-term success rate is high, we sometimes experience laborious cases with acute failure, recurrence, and prolonged procedural time^3,4^. The anatomy of the CTI is delineated by the borders of the tricuspid valve and the Eustachian ridge, it has been well examined in autopsies^5^. This anatomy is not consistent in all humans, and several studies have argued that CTI anatomy influences ablation results^6–9^. Based on previous reports, concave-shaped and/or pouch-like CTIs were related with more difficult procedures compared with straight-shaped CTIs. However, why CTI ablation is more difficult in concave CTIs has not been well elucidated.

Ablation with a transmural lesion is reasonable to achieve complete conduction block, and excessive atrial ablation must be avoided to reduce serious complications. Therefore, appropriate selections of ablation power, duration, and contact force are incremental for an optimal procedure. Recently, the ablation index (AI) has replaced force time integral (FTI) as a novel marker on a 3-dimensional (3D) mapping system (CARTO, Bioscence Webster, Inc, Diamond Bar, CA, USA)^10,11^. Especially in patients with atrial fibrillation, AI-guided ablation has been proven to be a more effective tool for pulmonary vein isolation because of its accuracy in estimating ablation lesion area compared with FTI. In consistency with atrial fibrillation, AI has recently been considered useful for guidance of CTI ablation^12^. At present, we are able to assess objectively and precisely each ablation lesion due to AI.

Deep pouch has been acknowledged as a predictor for laborious CTI ablation. Here, we investigated the relationship between CTI depth and first-pass success of CTI ablation, and then defined the cut-off value. In addition, we focused on the influence of AI on procedural results in comparison of shallow and deep CTIs.

## Material and methods

### Study design

All consecutive patients who underwent CTI linear ablation at Wakayama Medical University Hospital from January 2018 to December 2020 were retrospectively included in this study. Of these, a 3D electroanatomical mapping system (CARTO, Biosense Webstar, Irvine, CA) was not applied in three patients, computed tomography was not performed prior to CTI ablation in two patients, and ablations for recurrent AFL were performed in three patients. Therefore, these patients were excluded from the analysis. This study was carried out in accordance with the Declaration of Helsinki. This study was approved by the local ethics committee (Research Ethics Committee of Wakayama Medical University, 3053) and the requirement for written informed consent was waived because of the retrospective nature of the study.

### Ablation method

The patients were mildly sedated with hydroxyzine pamoate and/or dexmedetomidine hydrochloride. Right internal jugular vein access was obtained, and a multipolar deflectable catheter was inserted into the coronary sinus. Another multipolar catheter (Snake, Japan Life Line, Tokyo) was placed close to the tricuspid annulus via the right femoral vein. A deflectable sheath (Agilis, Abbott, St. Paul, MN) was also introduced from the right femoral vein for an ablation catheter (SmartTouch SF, Biosence Webster, Irvine, CA).

Radiofrequency ablation was performed starting from the tricuspid valve to the inferior vena cava (IVC). An irrigated ablation catheter was used with point-by-point application. In patients with AFL during the procedure, CTI-dependent AFL was diagnosed by entrainment pacing from the CTI. If the patient presented with sinus rhythm, continuous pacing at 500–700 ms cycle from the proximal coronary sinus was performed during CTI ablation.

**Figure 1:**
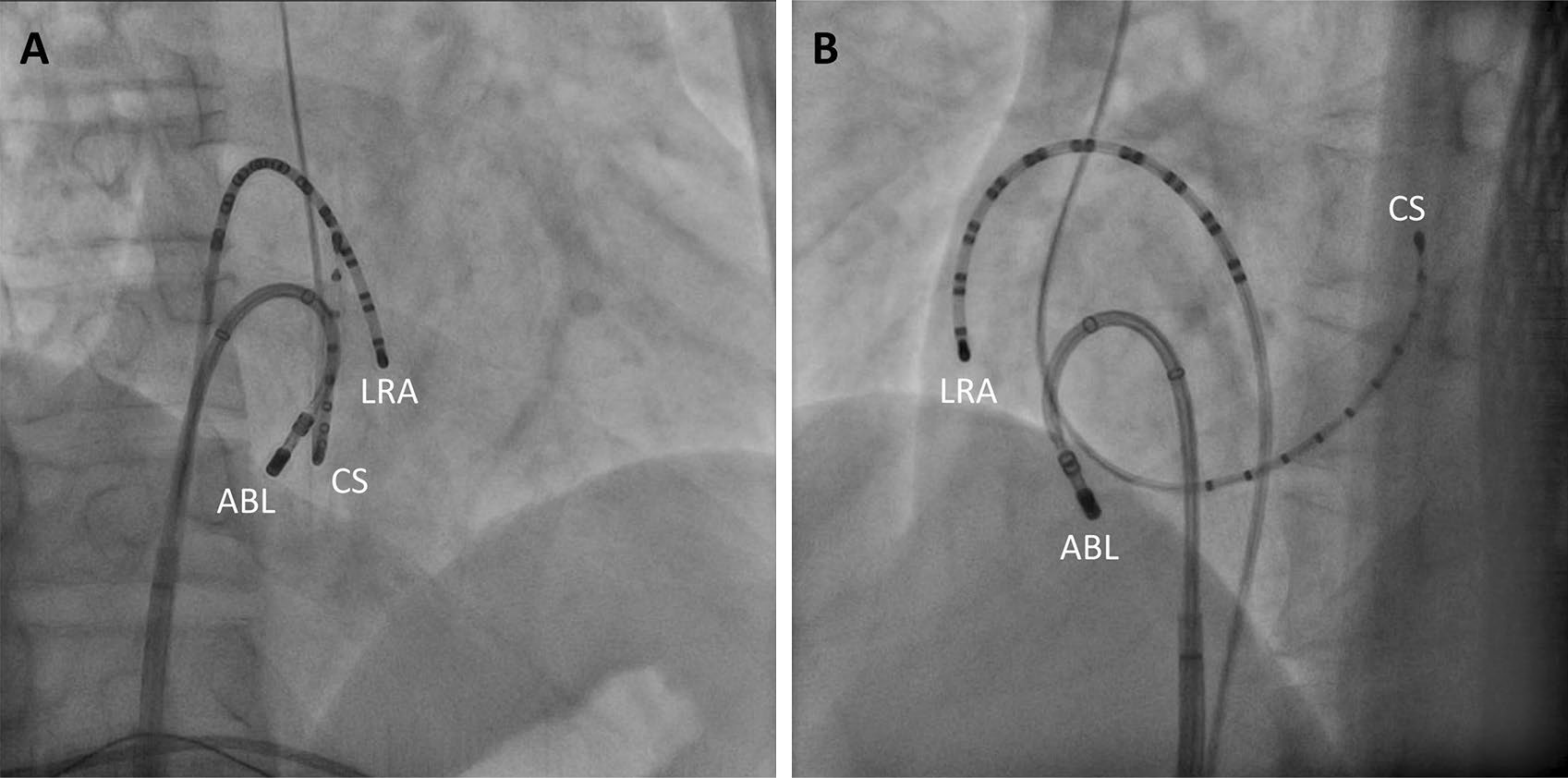
Catheter inversion technique. The ablation catheter is inverted to approach the Eustachian ridge. (**A**) Right anterior oblique. (**B**) Left anterior oblique. ABL, ablation catheter; CS, coronary sinus; LRA, lateral right atrium.

Both the VisiTag module and the fluoroscopy provided each radiofrequency (RF) location during the procedures. In this study, our setting of VisiTag is as follows: minimum time of 5 s, maximum range of 2.5 mm, minimum contact force (CF) of 5 g, and force over time of 25%. RF energy was 25–35 W, with saline irrigation at a flow rate of 8 mL/min or 15 mL/min, duration at each RF site was between 15 and 35 s, aiming for bipolar electrogram voltage attenuation of ≥ 80%, and distance between two neighboring RF sites did not exceed 6 mm. In cases with strong pain, 25 W of RF energy was applied, especially near IVC. Although AI data was displayed to operator in last 25 patients from April to December 2020, operators did not refer to AI during ablation and each AI data was retrospectively analyzed after ablation. The bidirectional conduction block was confirmed using differential pacing from the proximal coronary sinus and inferior lateral wall of the right atrium. The widely spaced double potential was also confirmed. If incomplete conduction block was suspected near the IVC, the ablation catheter would be deflected by more than 90° to ablate the interior of the pouch (Fig. 1)^13^. The complication was defined as vascular complication (pseudoaneurysm, fistula and bleeding requiring transfusion), cardiac tamponade, valvular damage, pneumothorax and hemothorax.

### Ablation data analysis

The RF sites of the CTI were divided into two parts: the ventricular side (2/3 segment of CTI) and the IVC side (1/3 segment of CTI)^12^. First-pass success was defined as requirement of no additional ablation after performing the first CTI line. Each VisiTag data, including ablation duration, mean CF, FTI, and AI, were retrospectively analyzed. The lowest AI sites of the first-pass CTI line were determined in both the ventricular and IVC sides (Supplementary Figure 1). The interrupted ablation sites due to catheter instability were defined as ablation duration of less than 15 s and excluded from the lowest AI site of the first-pass line determination.

### Computed tomography protocol and analysis

All patients underwent multi-detector computed tomography (MDCT) before catheter ablation. MDCT was performed using a 320-slice MDCT (Aquilion ONE, Canon Medical Systems, Otawara, Japan), with retrospective ECG-gated scans. In the routine protocol of atrial fibrillation, the total iodine dose was 160 mg/kg. On occasion, the application of contrast media was avoided because of chronic kidney disease and/or on the operator’s judgment. The images during the atrial diastolic phase of the cardiac cycle (30–40% of the interbeat interval) were reconstructed in different planes on a workstation (Intuition Thin Client, TeraRecon, Durham, NC, USA). Anatomical information was analyzed in the sagittal plane across both the center of the tricuspid valve and the IVC.

**Figure 2:**
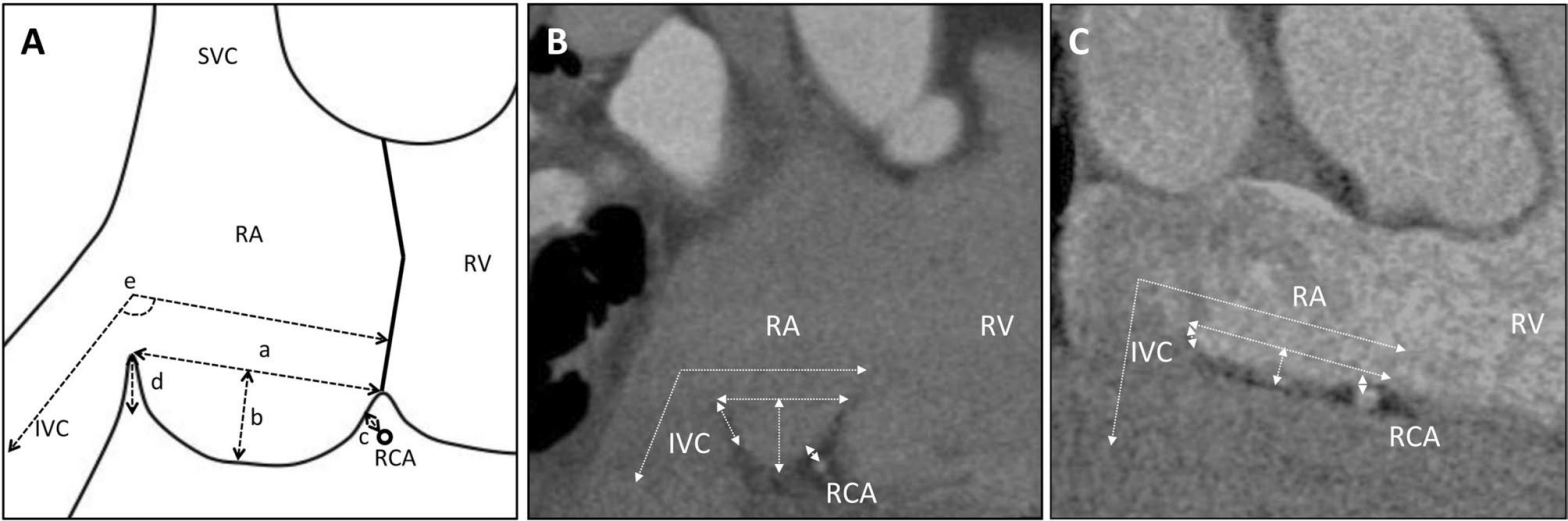
Measurement of cavotricuspid isthmus in computed tomography and representative cases. (**A**) Schema of quantitative measurement of the CTI. (**B**) The representative case with deep CTI. (**C**) The representative case with shallow CTI. CTI, cavotricuspid isthmus; SVC, superior vena cava; IVC, inferior vena cava; RA, right atrium; RV, right ventricle; RCA, right coronary artery.

As shown in Fig. 2, we analyzed the following: a) the length the CTI, b) the depth of the CTI, c) the length from the right coronary artery to the right atrium, d) the height of the Eustachian ridge, and e) the angle between the CTI and the IVC. The measurement of Eustachian ridge was made from its basis to its distal extremity in the plane passing through the center of the IVC^8^. In MDCT image with contrast media, the thickness of CTI was also analyzed. Measurement data of CTI was blinded to operators before ablation.

### Statistical analysis

Statistical analysis was performed using JMP Pro version 13.0 for Macintosh (SAS Institute, Cary, NC, USA). Results are expressed as median (interquartile range). Qualitative data are presented as numbers and percentages. The nonparametric Mann–Whitney U test was used to test for differences between the two groups. Pearson’s chi-square test was applied for categorical variables. The receiver operating curve (ROC) was used to determine the best cut-off value of CTI depth for first-pass success. The best cut-off value was determined according to maximum Youden’s index. A p value < 0.05 was considered statistically significant.

## Results

### Patient population

A total of consecutive 107 patients were enrolled in this study. Patient baseline characteristics, anatomical data collected by CT, and ablation results are summarized in Table 1. First-pass success was achieved in 60 (56%) patients. There were no major complications, but bidirectional block could not be achieved in one case. CTI ablation was performed during the AFL rhythm in 23 patients (21%) and AFL was terminated during ablation. In 73 patients (68%), 25 W of RF energy applied. CTI depth was significantly correlated with Eustachian ridge height (R^2^ = 0.30, *p* < 0.01). The thicknesses of CTI were evaluable in 43 patients with contrast enhanced MDCT and not significantly different between patient with and without first-pass CTI ablation (2.3 [2.1–2.6] mm vs 2.6 [2.1–3.0], *p* = 0.21).

**Table 1:** Patients' characteristics, anatomical data and ablation results.

**Patients characteristics**	
Patient, n	107
Age (years)	68 ± 12
Men	76 (71)
BMI, kg/m^2^	22.7 [20.9–25.0]
History of atrial fibrillation	62 (58)
Prior cardiac surgery	19 (18)
Left ventricular ejection fraction, %	57.3 [51.8–60.7]
Left atrial diameter, mm	39.0 [35.5–43.0]
More than moderate tricuspid regurgitation	10 (9)
**Anatomical data collected by CT**	
Length of CTI, mm	28.2 [24.3–33.1]
Depth of CTI, mm	4.6 [2.6–7.1]
CTI-IVC angle, degree	103.7 [93.1–114.5]
Height of Eustachian ridge, mm	1.5 [0.0–4.3]
Distance to right coronary artery, mm	2.5 [1.9–3.4]
Application of contrast media	58 (54)
**Ablation results**	
Bidirectional conduction block at the CTI	106 (99)
First pass success	60 (56)
Flutter at the start of the procedure	23 (21)
Complication	0 (0)

Data presented are median (interquartile range) or No.(%).

BMI: body mass index, CT: computed tomography.

CTI: cavotricuspid isthmus, IVC: inferior vena cava.

### CTI depth for first-pass success of ablation

**Figure 3:**
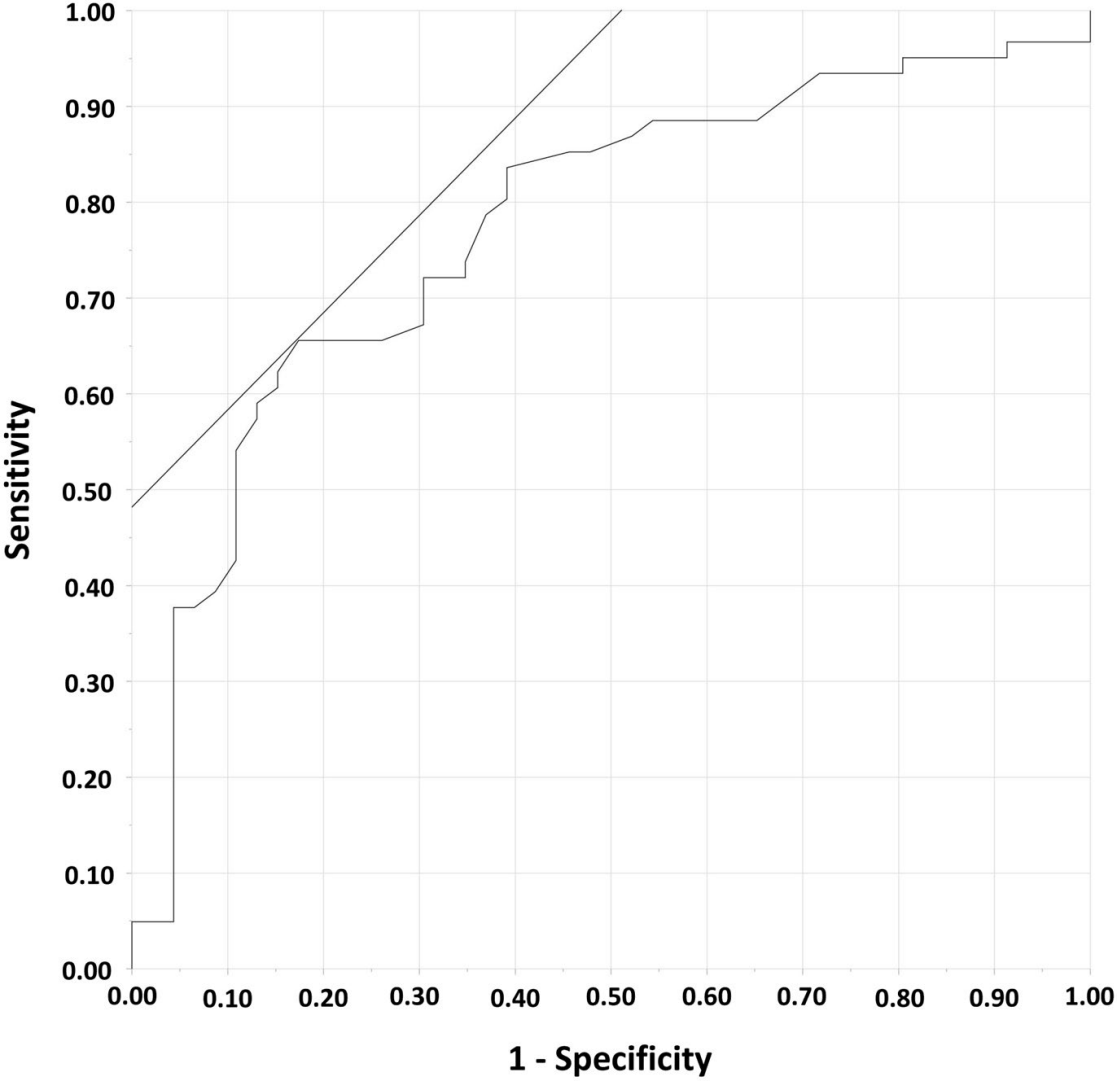
Receiver operating curve to predict first-pass success of CTI linear ablation according to the CTI depth. The area under the curve was 0.77 with a cut-off value of < 4.1 mm. The sensitivity and specificity, positive predictive value and negative predictive value were 67%, 81%, 82% and 66% respectively. CTI, cavotricuspid isthmus.

From the ROC, the best cut-off value of CTI depth to predict first-pass success of CTI ablation was < 4.1 mm with a sensitivity of 67% and specificity of 81% (Fig. 3).

### Ablation results

Catheter ablation procedural data in comparison with shallow CTI (SC; CTI depth < 4.1 mm) and deep CTI (DC; CTI depth ≧4.1) are summarized in Table 2. Although the CF at IVC side was higher in the DC than in the SC (*p* = 0.04), the DC required a longer ablation time (*p* < 0.01) and showed a lower first-pass success rate (*p* < 0.01). In addition, the catheter inversion technique was more frequently performed in the DC (*p* < 0.01). The global AI was not different between SC and DC (428.7 [411.6–44.3] vs 425.6 [404.0–438.1], *p* = 0.18).

**Table 2:** Procedural data.

	Shallow CTI	Deep CTI	*p* value
Number	49	58	
Ablation time	8 [6–11]	13 [9–20]	< 0.01
CTI block at first pass	40(81)	20 (34)	< 0.01
Catheter inversion technique	3(6)	33(57)	< 0.01
**RF time per application, s**			
Global	28.4 [26.7–29.4]	28.2 [26.4–29.2]	0.56
Ventricular side	29.4 [27.3–29.6]	28.3 [27.0–29.4]	0.25
IVC side	28.4 [25.5 -29.4]	26.8 [24.8–29.4]	0.21
**Contact force, g**			
Global	10.5 [9.4–12.0]	10.9 [9.7–12.4]	0.27
Ventricular side	10.9 [9.0–12.5]	10.6 [9.4–12.6]	0.98
IVC side	10.0 [8.5–11.5]	11.1 [9.1–14.0]	0.04
**Power, W**			
Global	29.0 [28.1–30.0]	28.4 [27.2–30.2]	0.11
Ventricular side	30.0 [29.4–31.3]	29.6 [28.5–30.6]	0.06
IVC side	26.9 [25.0–30.0]	26.5 [25.0–29.5]	0.24
**FTI, gs**			
Global	278.7 [249.0–341.6]	297.3 [256.5–353.8]	0.39
Ventricular side	271.5 [248.3–356.5]	290.2 [251.9–332.8]	0.96
IVC side	267.0 [232.7–306.3]	290.6 [238.3–375.8]	0.15
**Ablation index**			
Global	428.7 [411.6–444.3]	425.6 [404.0–438.1]	0.18
Ventricular side	440.4 [420.9–452.3]	432.9 [411.5–449.4]	0.14
IVC side	403.5 [390.7–419.0]	402.0 [379.3–419.1]	0.60

Data presented are median [interquartiles] or No. (%).

CTI, cavotricuspid isthmus; RF, radiofrequency; IVC, inferior vena cava; FTI, force time integral.

**Figure 4:**
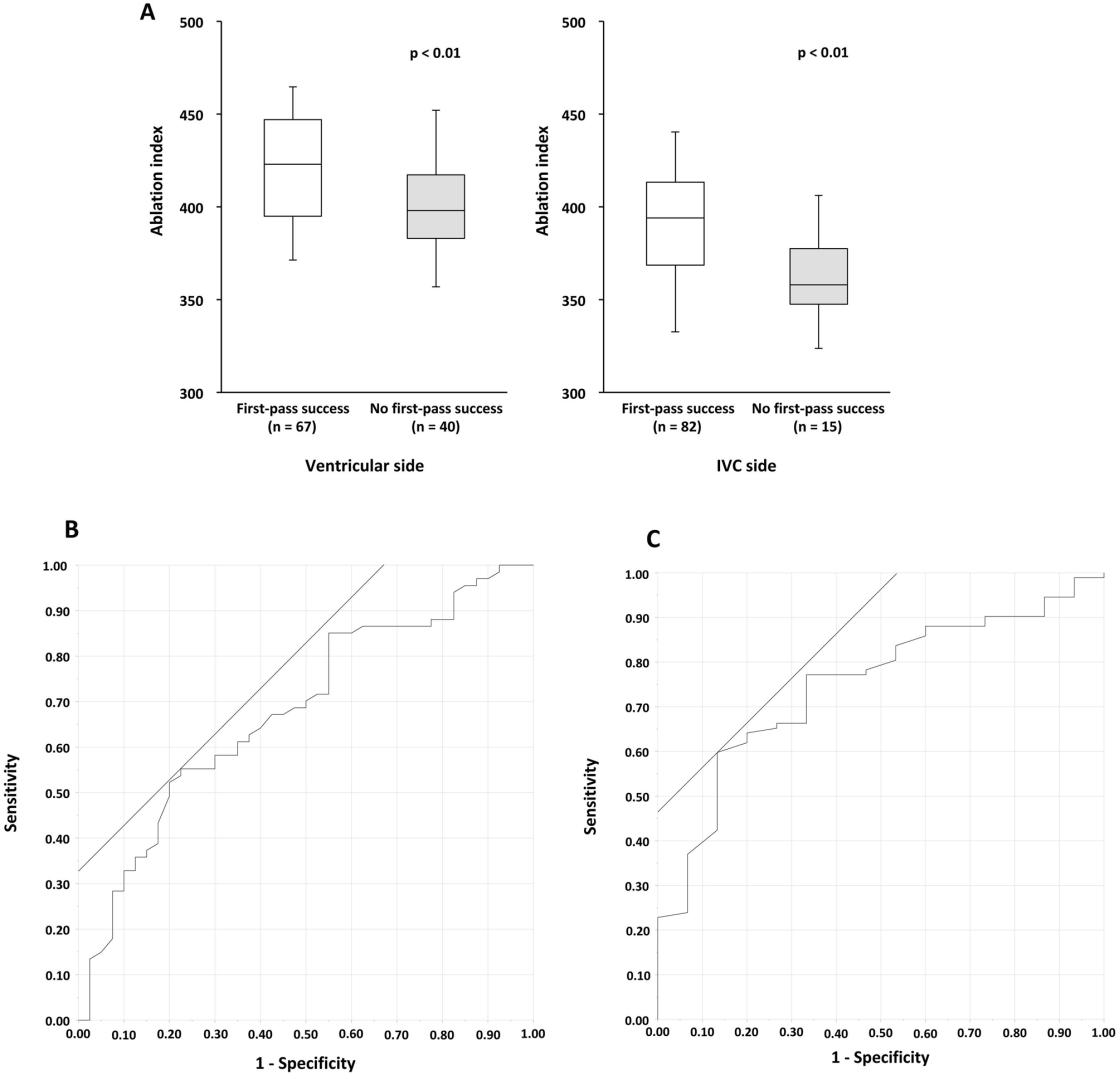
AI and first-pass success. (**A**) Box plot of lowest AI in comparison with first-pass success and no first-pass success cases. The lowest AI was higher in first-pass success cases both in ventricular side (*p* < 0.01) and IVC side (*p* < 0.01). Data are presented as box plots with median and 25th and 75th percentiles (box) and 5th to 95th percentiles (whiskers). IVC; inferior vena cava. (**B**) ROC for predicting first-pass success of CTI ablation according to the lowest AI at the ventricular side. The area under the curve was 0.68 with a cut-off value of > 420. The sensitivity, specificity, positive predictive value and negative predictive value were 55%, 78%, 80% and 51%, respectively. (**C**) ROC for predicting first-pass success of CTI ablation according to the lowest AI at the IVC side. The area under the curve was 0.75 with a cut-off value of > 386. The sensitivity and specificity were 60% and 87%. The sensitivity, specificity, positive predictive value and negative predictive value were 60%, 87%, 96% and 26%, respectively. AI, ablation index; CTI, cavotricuspid isthmus; ROC, receiver operating curve.

In both ventricular and IVC sides, averages of lowest AI were lower in unsuccessful cases than in successful cases (*p* < 0.01 and *p* < 0.01, respectively) (Fig. 4A). The conduction gaps in ventricular side (n = 40) were more frequently observed than in IVC side (n = 15) (37% vs. 14%, *p* < 0.01). From the ROC, the cut-off values of AI for predicting first-pass success of conduction block in ventricular and IVC sides were > 420 and > 386, respectively, with area under the curve of 0.68 and 0.75, respectively (Fig. 4B, C). Among patients with anterior (> 420) and posterior (> 386) AIs, first pass success was achieved in 16 of total 18 patients with SC and in 6 of total 12 patients with DC. Limited in patients with these cut off values, the first-pass success rate was higher in the SC than in the DC (89% vs. 50%, *p* < 0.05). The sensitivity and specificity, positive predictive value and negative predictive value of lowest AI cut off values to predict first-pass success were 40%, 78%, 89% and 23% in SC and 30%, 84%, 50% and 70% in DC, respectively.

## Discussion

### Major findings

In the present study, to best our knowledge, we firstly investigated the association of CTI depth and AI with first-pass success of radiofrequency catheter ablation for typical AFL. A CTI depth of < 4.1 mm was a best cut off value for first-pass success of CTI ablation. Although the individual ablation parameters were not different, the first-pass success rate was higher in the SC than in the DC.

### CTI depth and first-pass success

CTI ablation is an established strategy for typical atrial flutter and can provide durable success and a low recurrence rate. However, we sometimes experience laborious cases with prolonged procedural time. Considering several reports, the anatomy of the CTI can reflect procedural difficulty, and concave-shaped and/or pouch-like CTI, are especially associated with laborious interventions^6,7^. In this study, CTI of less than 4.1 mm depth was a best cut off for the first-pass success of CTI ablation. This cut-off value is similar to the < 3.9 mm CTI depth necessary for knuckle curve ablation reported by Shimizu et al.^13^. Consistent with a previous report, longer procedural time was observed in the deep CTI cases^7^.

### Ablation parameters in shallow and deep CTI

It has been acknowledged that CTI ablation tends to be more difficult in concave CTIs. However, it was unclear whether the CF was adequate, there was any gap between each ablation lesion, and ablation power was enough to achieve block line in concave CTIs. Currently, the CF and VisiTag module have been proven as effective tools, and the AI can evaluate lesion formation more precisely than FTI regardless of ablation power^14–17^. Although each ablation parameter was not different between the SC and the DC, the first-pass success rate of the SC was higher than that of the DC. In the present study, we conducted CTI ablation with a 3D mapping system, and there was no gap between each ablation point on the CARTO system. However, in deep CTIs, complete block line was not occasionally achieved regardless of the lesions having adequate AI.

Several potential mechanisms have been proposed. First, there might be a micro pouch, which was not assessed by CT, especially in the DC (Supplementary Fig. 2A). Second, the atrial wall in some patients may be thick. Knecht et al. reported that the thickness of the CTI is an independent predictor of difficulty in CTI ablation, but this could not be fully evaluated in this study because of non-contrast computed tomography (CT) imaging used in some patients^8^. Third, in patients with a high Eustachian ridge, a catheter inversion technique was required. Therefore, in this type of patients, it is difficult to achieve complete ablation of the lesions near the IVC (Supplementary Fig. 2B). Lastly, there might be a gap in the atrial myocardium regardless of the absence or presence of gaps in the 3D mapping system. The locations are result of the ablation catheter pushing the myocardium wall. Therefore, the curve shaped atrial wall in the DC might cause the gap (Supplementary Fig. 2C).

To improve the first-pass success rate, one of the strategies is to raise the target AI. In a previous report, the anterior and posterior AI targets were set at 500/400^12^. A higher first-pass rate of 93% was achieved without any complications. The appropriate target AI was decided according to the arterial wall thickness. Another solution is to modulate the catheter shape in the DC according to preoperative MDCT in formation although radiation exposure increases. Kajihara et al. adapted the procedure to anatomical characteristics by inverting the catheter near the IVC^18^. Combining the above two strategies, a tailor-made ablation method would be preferable by assessment of CT prior ablation. Further investigation is needed to improve AI guided CTI ablation, especially in deep CTI case.

### Study limitations

There are some limitations to this study. Because patients were retrospectively enrolled and derived from a single center, a selection bias may exist. Furthermore, the sample volume was relatively small. In addition, the CTI lines between actual ablation lesions and CT imaging might be different. Because this study focused only on the first-pass success rate of CTI ablation, its long-term efficacy remains uncertain. The data of catheter angulation was lacking. The thickness of the CTI was analyzed in limited populations, which could influence the success rate. In this study we divided CTI into ventricular and IVC side. Because we evaluate RF location based on VisiTag, the site might be different from anatomical assessment. Lastly, the area under the curve of ROC to predict first-pass success of conduction block in ventricular and IVC sides by AI was low.

## Conclusions

Although the ablation parameters were not different, the first-pass success rate was lower in the deep CTI than in the shallow CTI. Further investigation, including target AI, distance interval of RF sites and appropriate catheter position, would be required for better outcomes in deep CTIs.

## Supplementary Information

Supplementary Figures.
