# Supplementary material for: Impact of cavotricuspid isthmus depth on the ablation index for successful first-pass typical atrial flutter ablation
Source: Sci Rep. 2021 Nov 17;11:22413. doi: 10.1038/s41598-021-01846-7 (PMC8599492; doi:10.1038/s41598-021-01846-7)

Supplementary Figure 1. How to decide lowest AI sites of first-pass CTI

The RF sites of the CTI were divided into two parts: the ventricular side (2/3 segment of CTI) and the IVC side (1/3 segment of CTI). Then, lowest AI sites were selected from both ventricular and IVC side. In this case, 377 of AI in ventricular side and 372 of AI in IVC side were lowest.

AI: ablation index, CTI: cavotricuspid isthmus, IVC: inferior vena cava

Supplementary Figure 1

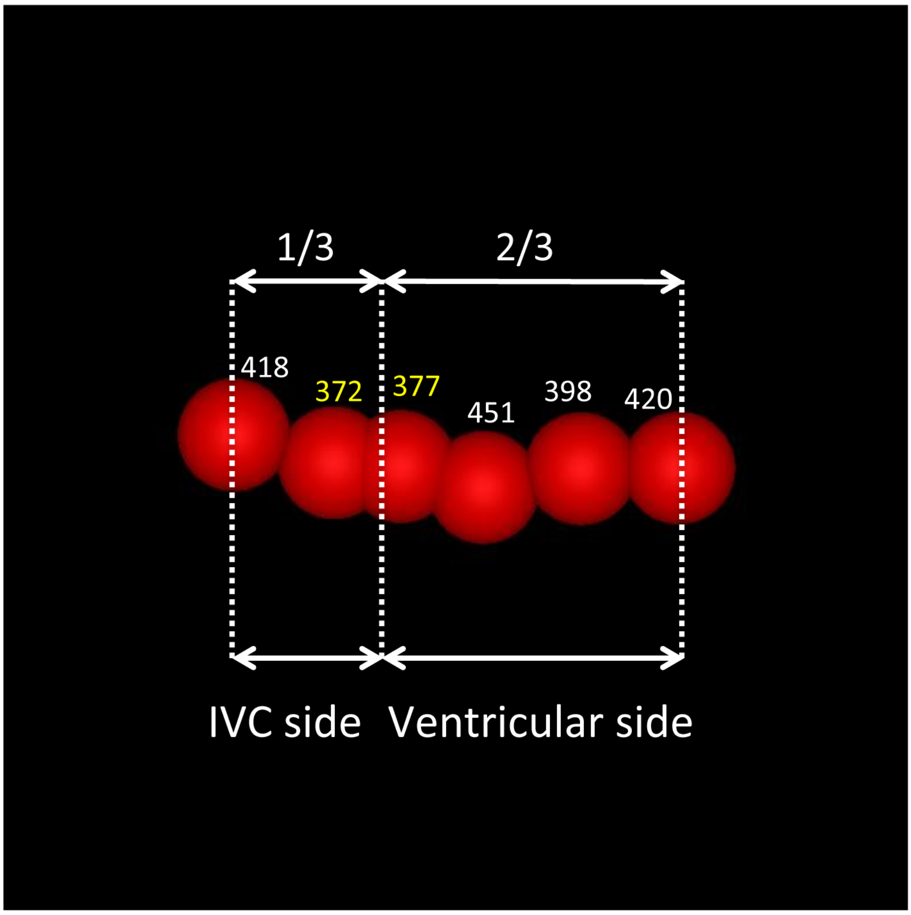

Supplementary Figure 2. Potential mechanism of procedural difficulty

A. Existence of micro pouch

B. Influence of Eustachian ridge

C. Potential of gap between ablation lesions

Supplementary Figure 2

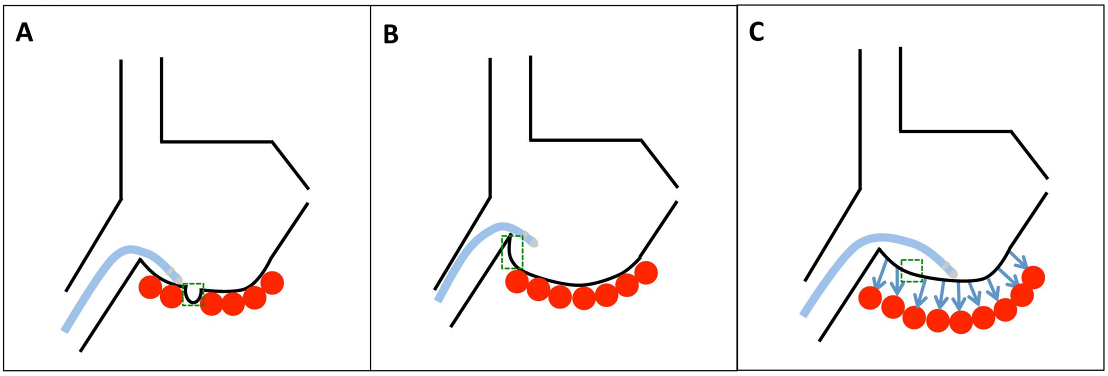

Supplement: Supplementary file 1 — Supplementary Figures. [file 41598_2021_1846_MOESM1_ESM.pdf]
